# Parkinson’s disease LRRK2 mutations dysregulate iron homeostasis and promote oxidative stress and ferroptosis in human neurons and astrocytes

**DOI:** 10.1186/s13024-026-00979-5

**Published:** 2026-07-24

**Authors:** Adamantios Mamais, Richard D. Batchelor, Aravindraja Chairmandurai, Thomas B. Ladd, Austin J. Shute, Nitya Subrahmanian, Nunziata Maio, Christopher D. Vulpe, Matthew J. LaVoie

**Affiliations:** 1 Center for Translational Research in Neurodegenerative Disease, Fixel Institute for Neurologic Diseases, Department of Neurology, University of Florida, Gainesville, FL USA; 2 Eunice Kennedy Shriver National Institute of Child Health and Human Development, NIH, Bethesda, MD USA; 3 Center for Environmental and Human Toxicology, Department of Physiological Sciences, College of Veterinary Medicine, University of Florida, Gainesville, FL USA

**Keywords:** LRRK2, Parkinson’s disease, Iron, lysosomes, Ferroptosis, Rab8a, iPSC, Neurons, Astrocytes

## Abstract

**Background:**

Iron accumulation is a hallmark of sporadic and familial Parkinson’s disease (PD) and correlates with clinical motor symptom severity. The biochemical mechanisms driving iron dyshomeostasis in PD brain and whether these are early or late events in the neurodegenerative process remain unknown. Nigral iron levels in LRRK2-PD patients have been reported to be even higher than in idiopathic PD, and greater in non-manifesting LRRK2 carriers than controls, suggesting that iron accumulation precedes clinical onset in LRRK2-associated PD. However, the cells affected and mechanisms governing iron dyshomeostasis in PD remain unclear.

**Methods:**

Here, we investigated multiple independent measures of iron homeostasis in human iPSCs and iPSC-derived models, including glutamatergic and dopaminergic neurons, and astrocytes, carrying pathogenic LRRK2 mutations. High-content and super-resolution microscopy of iron-specific probes and ICP-MS were used to determine iron content and distribution across different cell types and LRRK2 genotypes. Upstream effectors and downstream consequences of iron dyshomeostasis, including oxidative stress and ferroptosis-related phenotypes were also examined.

**Results:**

Pathogenic heterozygous LRRK2 mutations were associated with altered iron homeostasis across multiple human cell models. Lysosomal ferrous iron was consistently elevated across iPSC-derived glutamatergic neurons, dopaminergic neurons, and astrocytes carrying LRRK2 mutations and these phenotypes were reduced by varying degrees following treatment with the selective LRRK2 inhibitor, MLi-2. Importantly, we show that lipid peroxidation and ROS levels are elevated in isogenic LRRK2 mutant neurons, while iron chelation or MLi-2 reduced LRRK2-dependent ROS damage. In addition, CRISPR/Cas9-mediated loss of Rab8a produced overlapping iron-related phenotypes, while exogenous Rab8a expression normalized lysosomal iron levels in R1441C LRRK2 iPSCs, supporting a contributory role for Rab8a-dependent trafficking in these effects.

**Conclusions:**

Together, our findings show that pathogenic LRRK2 mutations converge on disrupted iron homeostasis across multiple human neural cell types, including dopaminergic neurons, and support a link between LRRK2 signaling, lysosomal iron dysregulation and ferroptosis-related stress.

**Supplementary Information:**

The online version contains supplementary material available at https://doi.org/10.1186/s13024-026-00979-5.

## Background

Iron accumulation in the substantia nigra is a well-recognized pathological hallmark of Parkinson’s disease (PD), observed in both sporadic and familial forms of the disorder and in other neurodegenerative conditions [1–7]. Iron overload correlates with neuromelanin loss in post-mortem PD brains [8] and is detectable in vivo through advanced MRI techniques, such as quantitative susceptibility mapping (QSM), where iron deposition in the substantia nigra pars compacta (SNc) has been correlated with motor symptom severity [9]. Notably, iron-sensitive imaging aids in distinguishing PD from other parkinsonian syndromes such as multiple system atrophy (MSA) and progressive supranuclear palsy (PSP) [10–14]. Despite decades of evidence linking iron dyshomeostasis to PD, the molecular underpinnings that drive brain iron accumulation and neuronal vulnerability and whether these are early or late features of disease remain incompletely understood.

Iron’s potential to promote oxidative stress through Fenton chemistry and the generation of reactive oxygen species (ROS) make it a likely contributor to dopaminergic (DA) neuron degeneration [15, 16]. Ferroptosis, a regulated form of iron-dependent cell death, has emerged as a key mechanism linking iron overload to neuronal loss [17–19] and has been widely implicated in PD [20–24], but how genetic factors for PD intersect with ferroptotic pathways is not fully defined. Mutations in leucine-rich repeat kinase 2 (LRRK2) are the most common cause of familial PD [25] and have been implicated in vesicular trafficking defects and lysosomal dysfunction [26–32], processes that could plausibly disrupt iron handling. Indeed, clinical imaging studies reveal that individuals carrying pathogenic LRRK2 mutations, whether symptomatic or asymptomatic, exhibit elevated nigral iron levels compared to idiopathic PD patients and healthy controls [33], suggesting that iron dyshomeostasis could be an early and contributing feature in LRRK2-associated PD. However, the cellular and molecular mechanisms underlying these clinical observations remain unknown.

Autosomal dominant missense mutations in LRRK2 cause a late-onset form of familial PD while genome-wide association studies have identified risk-factor variants of LRRK2 linked to idiopathic PD [25]. PD-linked mutations segregate within the kinase domain (e.g. G2019S) and the Roc-COR tandem domain (e.g. R1441C, Y1699C) of LRRK2 and increase phosphorylation of over a dozen Rab GTPase substrates at a conserved threonine/serine residue [34, 35], thereby altering Rab-effector interactions and intracellular trafficking, with downstream consequences for endolysosomal dynamics [26–29]. Given that Rab GTPases govern critical steps in membrane trafficking and receptor recycling [36], these pathways offer a potential intersection between LRRK2 function and iron regulation. In our previous work, we observed alterations in trafficking of the transferrin receptor (TfR), that mediates iron uptake in most cell types [37–40], in mutant LRRK2 overexpression models [32]. Importantly, we reported an increase in brain ferritin and iron load in homozygous G2019S KI mice compared to WT controls in acute pro-inflammatory conditions [32]. Whether such disturbances occur basally and across different LRRK2 mutations, particularly in human neurons and glia when expressed in the heterozygous state, remains unclear. Astrocytes are key regulators of brain iron levels and availability and are implicated in PD neuropathology [41–47]. Furthermore, LRRK2 expression is enriched in astrocytes and we and others have reported effects of LRRK2 mutations on astrocytic function [48, 49]. How endogenous LRRK2 mutations specifically affect cellular iron status and distribution in human astrocytes and neurons as well as the role of altered iron in neuronal damage remain unexplored.

In this study, we leverage patient-derived and isogenic induced pluripotent stem cell (iPSC) models to investigate how pathogenic LRRK2 mutations influence iron regulation in human neurons and astrocytes. By examining a wide panel of iron-related phenotypes, our data revealed convergent effects of heterozygous R1441C, Y1699C and G2019S LRRK2 mutations on intracellular and lysosomal iron content that were rescued by LRRK2 inhibition. Furthermore, we report distinct effects of LRRK2 mutations on ferritin levels and a concomitant effect on basal Iron Regulatory Protein Iron-Responsive-Element (IRE)-binding activity, suggesting alterations in ferritin expression and/or post-transcriptional regulation. Pathogenic LRRK2 kinase activity is thought to alter Rab8a function, which, in our previous work, showed a special relationship with cellular iron [32]. Here, genetic Rab8a ablation phenocopied selected iron-related defects observed in LRRK2 mutant cells, while exogenous Rab8a expression normalized lysosomal iron levels in heterozygous R1441C LRRK2 cells. Basal ROS levels and lipid peroxidation were elevated in LRRK2 mutant neurons and reduced by iron chelation or LRRK2 kinase inhibition. Importantly, kinase-dependent increases in lysosomal iron and lipid peroxidation were observed in LRRK2-mutant dopamine (DA) neurons, supporting the engagement of ferroptotic signaling in this cellular model of PD. Our data highlight a discrete pathway affected by heterozygous pathogenic LRRK2 mutations that results in the misregulation of this transition metal, leading to cellular injury. These data suggest that iron dyshomeostasis may represent one mechanism linking pathogenic LRRK2 signaling to ROS reported across different PD models [50–52].

## Methods

### Induced pluripotent stem cell (iPSCs) cultures

We used iPSC lines carrying heterozygous LRRK2 mutations from three independent sources.

BR33 were derived from a Caucasian donor, who was deeply phenotyped as part of the ROS/MAP longitudinal aging studies [53, 54] and determined to not be cognitively impaired at death at age > 89 and free from genetic variants that confer risk of PD. ND35371 (R1441C heterozygous) and ND50051 (G2019S heterozygous) were obtained by the NINDS Repository. Five G2019S heterozygous, two R1441G heterozygous and five WT iPSC lines were obtained from the Parkinson’s Progression Markers Initiative dataset (PPMI, ppmi-info.org) along with longitudinal clinical data. The isogenic mutant LRRK2 lines (all heterozygous mutations, homozygous KO) were a kind gift from Dr. Mark Cookson (NIA, NIH) [55].

## CRISPR/Cas9 genome editing of iPSCs and HEK293 cells

Rab8a and Rab10 knockout lines were generated in iPSCs and HEK293 cells as described before [56]. Briefly, single guide RNAs (sgRNAs) were selected using a web-based design tool (http://crispr.mit.edu). Rab8a sgRNA: 5’ GAACTGGATTCGCAACATTG 3’; Rab10 sgRNA: 5’ ATGGCTTAGAAACATAGATG 3’. These were cloned into pXPR_003 (Addgene #52963) and sequenced using the primer 5′-GATACAAGGCTGTTAGAGAGATAATT-3′ to determine clones that successfully integrated the sgRNA. BR33 iPSCs were generated and characterized in collaboration with the New York Stem Cell Foundation (NYSCF) using described methods [54, 57, 58]. iPSCs were co-transfected with plasmids that express the sgRNA plasmid and a nuclease-active SpCas9 expression vector (Addgene #62988). After 2 days, cells that were successfully transfected with the two plasmids were selected by puromycin treatment for 4 days. Individual clones were identified by plating ∼1 cell per well in a 96-well dish and allowed to grow for 2 weeks. Monoclonal lines were identified, expanded, sequenced, and stocked. Amplification of Rab8a and Rab10 genes was conducted according to manufacturer’s protocol (Invitrogen K2030-01) and homozygous gene editing was confirmed by Sanger sequencing. Multiple sequence alignment was performed using ClustalW in BioEdit.

## iPSC differentiation into mature astrocytes

iPSCs were differentiated into iAs using the protocol developed by Canals and colleagues [59]. Briefly, iPSCs were maintained in mTeSR media and transduced with lentivirus to stably express pTet-O-NFIB (hygromycin), pTet-O-SOX9 (puromycin) and FUdelta GW-rtTA. Differentiation of pure human iA cultures was achieved by culturing transduced cells in doxycycline to induce expression of Sox9 and Nfib and to select against non-differentiated cells with selection media (puromycin and hygromycin). Human iAs were studied at day ~ 21 post-differentiation. iAs show marked expression of GFAP by staining and immunoblot and robust expression of LRRK2 and relevant Rab GTPases.

## Differentiation of NGN2-induced neurons

iPSCs were differentiated into NGN2-induced neurons (iNs), as before [29, 56, 60]. Briefly, iPSCs were cultured on Matrigel-coated plates in StemFlex (A33493) media and co-transduced with lentivirus packaged with pTet-O-NGN2-puro and Fudelta GW-rtTA plasmids (Zhang et al., 2013) for 2 days and passaged for expansion. NGN2-transduced iPSCs were thawed in StemFlex media with ROCK inhibitor (10 µM; Stemcell Technologies, 72304) for 24 h then plated at 2 × 10^6^ cells/10 cm plate in StemFlex and grown until 75% confluent. For differentiation, on day 1 cells were fed with KnockOut media (Gibco 10829.018) supplemented with KnockOut Serum Replacement (Invitrogen 10928-028), 1% MEM non-essential amino acids (Invitrogen 11140), 1% GlutaMAX (Gibco 35050061) and 0.1% βME (Invitrogen 21985-023) (KSR) with doxycycline (2 µg/ml, Sigma, D9891-5 g) to induce NGN2 expression. On day 2, they were fed with a 1:1 ratio of KSR: N2B media (DMEM F12 supplemented with 1% GlutaMAX, 3% dextrose and N2-Supplement B; StemCell Technologies 07156) with puromycin (5 µg/ml; Life Technologies, A11138-03) and doxycycline to select for transduced cells. On day 3, the cells were fed with N2B media with B27 (1:100; Life Technologies, 17504-044), puromycin, and doxycycline. On day 4, induced neurons (iNs) were frozen down in 10% DMSO/FBS in Neurobasal media (NBM Gibco 21103-049) supplemented with B27, BDNF (Peprotech, 450-02), CNTF (Peprotech, 450 − 13), and GDNF (Peprotech, 450 − 10) all at 100 ng/mL, ROCK inhibitor (10 µM), puromycin, and doxycycline. iNs were plated and grown in NBM with B27, BDNF, CNTF, GDNF, puromycin, and doxycycline until day 21.

## iPSC differentiation into DA neurons

iPSCs were cultured in StemFlex (A33493) media and co-transduced with lentivirus packaged with pTet-O-NGN2-puro and Fudelta GW-rtTA plasmids for 2 days and passaged for expansion, as before [29, 56, 60]. To begin the dopaminergic neuron differentiation, NGN2-transduced iPSCs were thawed in mTeSR Plus media (StemCell technologies,100–0276) with 10 µM ROCK inhibitor (StemCell Technologies, 72304) and plated at 2 × 10^6^ cells/10 cm plate coated with Matrigel (Corning, 354277) and grown to 75% confluency - considered Day − 1 (D-1). The iPSCs were differentiated into mature dopaminergic neurons using established protocols [61, 62]. Briefly, at D0 the cells were treated with media containing growth factors and 2 µg/mL Doxycycline (ThermoFisher, J6057914) (Dox; present through differentiation) to begin the differentiation and Puromycin (ThermoFisher, A1113803) was added to the formulation for D1. On D2, cells were plated in media containing growth factors and Dox with ROCK inhibitor at 5 × 10^4^ cells/well in 96 well plates (Greiner, 655090) coated with Poly-L-ornithine hydrobromide (Sigma-Aldrich, P3655) - Laminin (Sigma-Aldrich, CC095-M). STEMdiff Midbrain Neuron Differentiation (Kit) media (StemCell Technologies, 100 − 0038) was used on D3 & D4 supplemented with Dox, 1 µM Cytarabine (Sigma-Aldrich, 251010), and 200 ng/mL SHH (StemCell Technologies, 78065). Daily media changes continue D5-D8 in the presence of Dox and SHH. D9-D21, cells were cultured using STEMdiff Midbrain Neuron Maturation (Kit) media (StemCell Technologies, 100 − 0038) with half media changes every third day. Cells were imaged at D19-D21.

### PPMI UPDRSIII data

Data used in the preparation of this article were obtained from the Parkinson’s Progression Markers Initiative (PPMI) database (www.ppmi-info.org/access-data-specimens/download-data), RRID: SCR_006431. Openly available UPDRSIII data for five G2019S and two R1441G mutations carriers were used (Tier 1 data downloaded between 03 November 2023 and 10 November 2023).

## IRE-binding activity assays

IRP activity was assessed using biotinylated IRE RNA probes, as previously described [63]. Briefly, probes were 3’-biotinylated and sourced from Eurofins Genomics or IDT. Cell lysates were prepared in lysis buffer (40 mM KCl, 25 mM Tris-Cl pH 7.5, 1% Triton X-100) with protease inhibitors (ThermoFisher #78430), 1 mM DTT, and 20 U/ml RNase inhibitor (ThermoFisher #AM2696). For binding, 5 pmol of annealed IRE probes were incubated with 100 µg DynaBeads M280 for 20 min at room temperature, washed three times, then mixed with 100 µg lysates for another 20 min. IRP-bound complexes were isolated magnetically, washed, and resuspended in 100 µL LDS buffer, denatured at 95 °C for 5 min, and analyzed by western blot (25 µL on 4–12% Bis-Tris gels) for IRP1 and IRP2.

## ICP-MS

iPSCs in culture were collected in ice-cold PBS and divided equally into two tubes for ICP-MS and biochemical analysis of protein levels, pelleted and frozen in dry ice. Frozen iPSC pellets were resuspended in 200 µl PBS and mixed with 200 µl of concentrated trace-metal-grade nitric acid (Fisher) in 15 ml Falcon tube. The samples were digested at 85 °C overnight, diluted to 4 ml with deionized water, and then analyzed by Agilent 7900 ICP-MS at the Analytical Toxicology Core Laboratory (ATCL) at the University of Florida. Metal levels were normalized to total protein content.

### Cell treatments

For iron overload or chelation, cells were treated with 150 µM ferric ammonium citrate (FAC) (SIGMA) or 100 µM of deferoxamine in normal media overnight prior to analysis by imaging or western blotting. For rescue experiments, cells were treated with 100 nM MLi-2 (Tocris) for 2 h or 7 days in culture prior to analysis. LLOMe was used at 1 mM for 3 h in culture.

### iPSC transfection

Human iPSCs were transiently transfected with GeneJuice (Millipore) at ~ 60–70% confluency using a 3:1 µL GeneJuice:µg DNA ratio prepared in Opti-MEM (ThermoFisher). DNA-GeneJuice complexes were added directly to cells in mTeSR1 medium, and transgene expression and cellular analyses were performed 24–48 h post-transfection. The constructs eGFP-Rab8a (Addgene #86075) [64] and pcDNA3.1 eGFP (Addgene #129020) were used.

### Western blot

Cells were lysed in ice-cold 1x Cell Signaling Lysis Buffer (Cell Signaling #9803S) supplemented with Halt phosphatase and protease inhibitor cocktail (Thermofisher Scientific), for 30 min on ice. Lysates were clarified at 21,000 x *g* for 10 min at 4 °C, and supernatants were quantified by Pierce 660 nm protein assay (22660). Samples were supplemented with NuPage LDS sample buffer 4x (#NP0008), denatured for 10 min at 65 °C and analyzed by standard western blot protocols using the following antibodies: FTH1 (Cell Signaling 4393 S), FTL (Abcam AB69090), TfR1 (13-6800), DMT1 (Abcam AB123085), Cyclophilin B (Abcam AB16045). Secondary LiCOR antibodies were used for detection using the LiCOR model M scanner. Uncropped blots are presented in Supplementary Fig. 10.

### Immunocytochemistry

Cells were seeded at 120,000/well (24-well plate) on 12 mm coverslips precoated with Geltrex basement membrane matrix (A1413201, Thermo) and cultured as described above. Cells were fixed in 4% (w/v) formaldehyde/PBS for 15 min, permeabilized in 0.2% Triton X-100/PBS for 10 min at RT, blocked in 5% (v/v) FBS in PBS, and incubated with primary antibodies (pT73 RAB10: MJF-R21-22–5, ab241060, Abcam; TH: CPC-TA, EnCor Bio) in 1% (v/v) FBS/PBS overnight at 4 C. Following 3 washes in PBS, the cells were incubated for 1 h with secondary antibodies (Alexa Fluor 488, 568, 647-conjugated; ThermoFisher). After 3 PBS washes, the coverslips were mounted, and the cells were imaged by confocal microscopy (Nikon CSU-W1-SORA).

### High-content imaging and quantitative analysis

For high-content live-cell imaging, cells were plated at 10,000 cells per well in 96-well black-wall clear-bottom plates (µClear, Greiner), labeled with FerroOrange (Dojindo), HMRhoNox-M (Lumiprobe), Liperfluo (Thermo), LysoTracker Red (Invitrogen) and CellROX (Thermo) according to the manufacturer’s specifications, and 20 ng/ml of Hoechst. CellLight Lamp1-emGFP BacMam 2.0 or LysoTracker (Thermo) were used to visualize lysosomes according to the manufacturer’s specifications. Labeled live cells were imaged using the Lionheart high-content automated microscope (Agilent) (10x or 20x objectives), or the ImageXpress HCS.ai high-content confocal imaging platform (Molecular Devices; 60x objective), collecting multiple fields per well for coverage of > 800 cells per well. The DAPI, GFP, RFP/TRITC and Cy5 channels were used and images were analyzed on the Gen5 software (Lionheart; Agilent) or IN Carta^®^ software (Molecular Devices). For experiments involving GFP control vector or GFP-Rab8a expression, quantitative analyses were restricted to GFP-positive cells to ensure consideration of successfully transfected cells. To account for plate-to-plate variability in staining intensity, fluorescence measurements were normalized to the corresponding control condition within each imaging plate before pooling data across biological replicates.

### Quantification of lysosomal iron using whole-cell, lysosome-restricted segmentation

Quantitative analysis of HMRhoNox-M fluorescence was performed using the IN Carta^®^image analysis software (Molecular Devices) using two complementary segmentation strategies. For whole-cell analysis, nuclei were identified using Hoechst staining and used as primary objects. Cytoplasmic regions were generated based on the primary objects (nuclei) using TRITC staining for cell perimeter, producing a whole-cell mask for quantification of integrated HMRhoNox-M signal on a per-cell basis. For lysosome-restricted analysis, lysosomes were identified using LysoTracker staining. LysoTracker-positive puncta were segmented as subcellular objects using intensity-based thresholding and size filtering, producing a lysosomal mask per cell (similar to [65]), and HMRhoNox-M signal was quantified within lysosomes. Measurements were aggregated across multiple fields per well, with > 800 cells analyzed per biological replicate.

### Imaris analysis

Following confocal microscopy, the Imaris platform (Oxford Instruments) was used to analyze the localization and distribution of lysosomal iron in lysosomes. Z-stack confocal images of iAs were processed through the Imaris Surface Contour module to render lysosomal iron, lysosomes and nuclear staining to surfaces and measure the distance between lysosomes and proximity to the nuclear edge throughout z planes in the 3D volume.

### Statistical analyses

All experiments were conducted at least three independent times for three differentiations. Error bars indicate mean ± SD. Statistical analysis was performed using GraphPad Prism software, using a one-way ANOVA with Dunnett’s post-hoc or two-way ANOVA with Tukey’s post-hoc test.

## Results

### Pathogenic LRRK2 mutations disrupt iron-related pathways in patient-derived and isogenic iPSCs and neurons

**Fig. 1:**
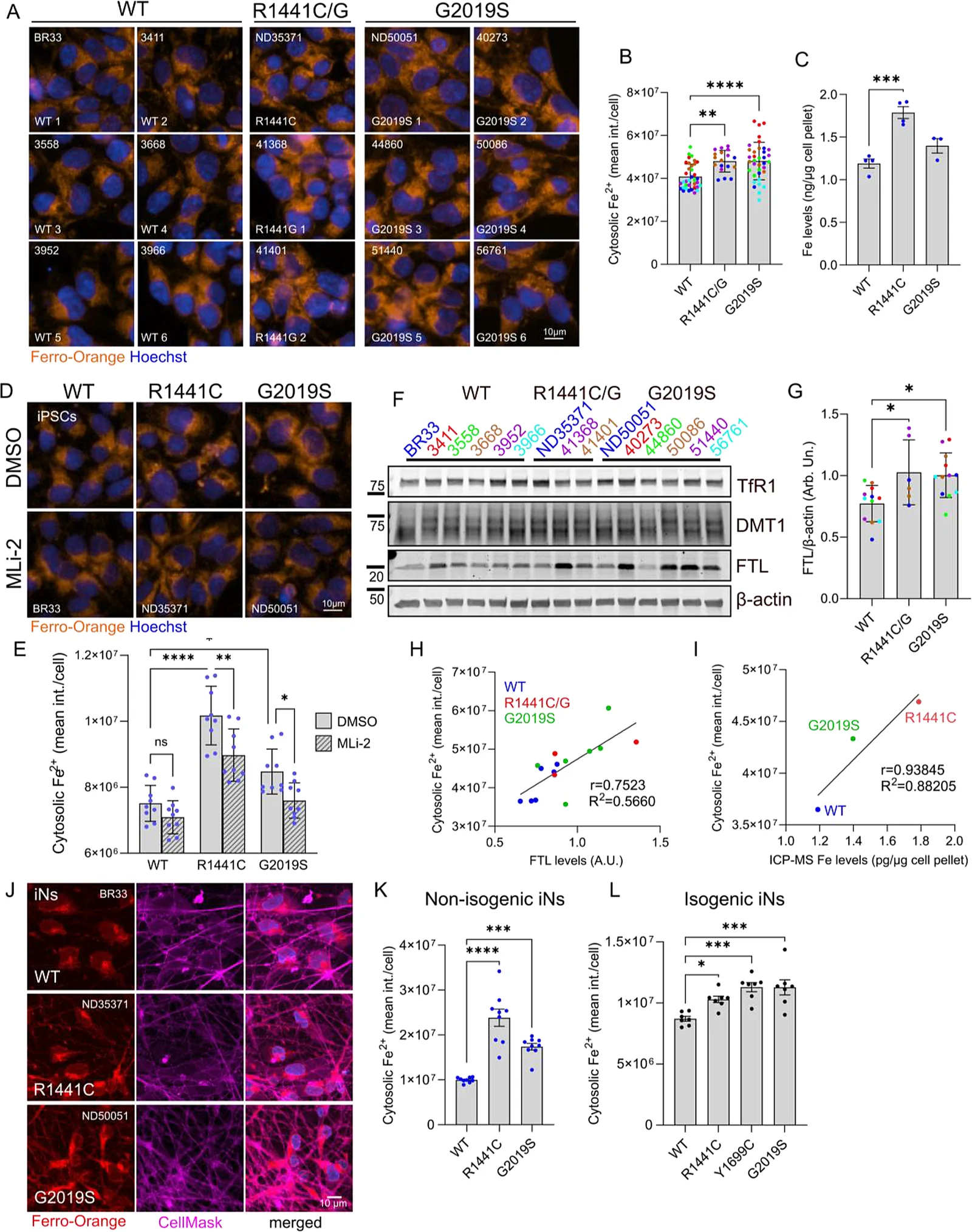
LRRK2 mutations alter iron-related phenotypes in human iPSCs and iNs. (**A, B**) Cytosolic free iron imaging and quantitation (FerroOrange) in six WT, six G2019S, one R1441C, and two R1441G LRRK2 iPSC lines (PPMI, NINDS Repository) (*N* = 6 wells each line; each line represented in a different color within each genotype group; one-way ANOVA, Dunnett’s post-hoc pair-wise comparison). (**C**) Quantitation of total iron in WT, R1441C and G2019S iPSCs (NINDS Repository) by ICP-MS. (**D, E**) Imaging and quantitation of cytosolic free iron (FerroOrange) in mutant LRRK2 iPSCs (NINDS Repository) following MLi-2 treatment (100 nM, 7 days) (two-way ANOVA, Tukey’s post-hoc; *N* = 9 biological replicates (> 800 cells per N), Genotype ****p = 0.0001, Treatment ****p < 0.0001, Interaction *p* = 0.2318). (**F, G**) Western blot and quantitation of levels of iron-related factors in WT, heterozygous R1441C/G, and G2019S iPSCs (*N* = 2 biological replicates per line; 6 WT, 2 R1441G, 1 R1441C and 6 G2019S lines, one-way ANOVA, Dunnett’s post-hoc; *p < 0.05). (**H**) Scatter plot showing a positive correlation between cytosolic iron (FerroOrange) and FTL levels (*r* = 0.7523). (**I**) Scatter plot showing a positive correlation between cytosolic ferrous iron levels (FerroOrange) and total cellular iron quantified by ICP-MS across iPSC lines. Each data point represents the mean value for a single iPSC line (WT: BR33, R1441C: ND35371, G2019S: ND50051), calculated from > 4 independent biological replicates for ICP-MS and 6 independent imaging wells for FerroOrange (> 800 cells per well). Pearson correlation shown. (*r* = 0.93845). (**J** to **L**) Cytosolic iron imaging and quantitation in mature iNs carrying heterozygous LRRK2 mutations (**J, K**) as well as isogenic iNs versus controls (NIA, NIH) (**L**) (one-way ANOVA, Dunnett’s post-hoc, *N* = 8 biological replicates, (> 800 cells per N), *p < 0.05, **p < 0.01, ***p < 0.001, ****p < 0.0001)

In our previous work, we identified a signature of altered iron pathways in the kidneys of homozygous G2019S knockin mice by unbiased proteomics, highlighting divergent expression of endolysosomal and mitochondrial factors involved in iron regulation in vivo [35]. Separately, we reported increased accumulation of iron in the brains of homozygous G2019S LRRK2 mice following inflammation compared to WT controls [32]. These data were the first to suggest that G2019S LRRK2 dysregulates aspects of intracellular iron homeostasis in LRRK2 models. To examine whether these pathways are affected by endogenous LRRK2 mutations beyond G2019S, and, when present in the heterozygous state most commonly associated with PD, we assessed multiple aspects of iron homeostasis in iPSC-derived models from LRRK2-PD patient samples. While the field has more commonly relied on other patient-derived cells such as fibroblasts [66] or differentiated neural derivatives, undifferentiated iPSCs have also been used to study mutant LRRK2 biology, for example, in ciliogenesis [67]. Here, we interrogated undifferentiated iPSCs as a simple human genetic context in which iron-related alterations may be detected. We quantified cytosolic iron levels in six WT, six G2019S, one R1441C, and two R1441G LRRK2 iPSC lines (all heterozygous) by high-content imaging of the FerroOrange probe, a fluorescent indicator of labile iron. We observed an increase in the steady-state labile ferrous iron pool across different LRRK2 mutant lines compared to WT lines (Fig. 1A, B). Each iPSC line is depicted in a different color to reflect the biological variability observed across the many patient-derived lines (Fig. 1B). Increased total iron loading in the R1441C LRRK2 mutant iPSCs was further confirmed by ICP-MS (Fig. 1C). Lastly, the small molecule LRRK2 kinase inhibitor MLi-2 reversed the increase in labile iron levels in heterozygous G2019S iPSC lines compared to WT, with a partial rescue in R1441C LRRK2 iPSCs (Fig. 1D, E). Next, we investigated proteins involved in iron import (TfR1, DMT1) and iron storage (ferritin light chain; FTL). We observed an overall increase in FTL levels across the R1441C/G and G2019S mutant lines compared to WT iPSC controls (Fig. 1F, G), while the levels of TfR1 and DMT1 were comparable between lines (Fig. 1F and Supplementary Fig. 1A, B). Despite an overall effect of mutations on iron-related readouts, we noted biological variability in the levels of labile iron (Fig. 1B) and ferritin (Fig. 1G) across the different patient-derived iPSC lines. We observed moderate correlation between labile iron levels (FerroOrange) and FTL levels (R^2^ = 0.5660, F(1,13), *p* = 0.0012) (Fig. 1H), while FerroOrange and total iron levels detected by ICP-MS trended in the same direction across the three lines tested (R2 = 0.8805, F(1,1), *p* = 0.2247) (Fig. 1I). We did not observe a correlation between ferritin levels in the iPSC lines and motor symptom severity (UPDRS III scores) in LRRK2 PD or control groups reported for the iPSC donors (Supplementary Fig. 1C).

Since iron dyshomeostasis is a key feature in PD pathology and our data support altered iron levels in LRRK2 mutant iPSCs, we next examined human iPSC-derived NGN2-induced neurons (iNs) to understand how this change translates in post-mitotic cells. iPSCs (NINDS Repository) were differentiated into iNs by forced expression of NGN2 via lentiviral gene delivery, according to established protocols [29, 56, 60] (Supplementary Fig. 2). We detected an increase in the labile iron pool across heterozygous R1441C and G2019S LRRK2 neurons compared to WT controls (Fig. 1J, K). Because donor-derived iPSC lines showed appreciable biological variability (Fig. 1B), these supportive data warranted further analysis in a series of isogenic LRRK2-mutant iPSCs [55].

These isogenic iPSC lines were generated by CRISPR/Cas9 in a single female WT iPSC background, A18945, with editing validated and clones subsequently characterized for morphology, karyotype and differentiation potential [55]. We characterized these cells by validating detection of endogenous LRRK2-dependent signaling. LRRK2 protein is detectable, albeit lowly expressed, across WT iPSCs and differentiated neuron and glial models used in this study (Supplementary Fig. 2C). Furthermore, we found multiple lines of evidence for endogenous LRRK2-dependent signaling (consistent with earlier studies in undifferentiated iPSCs [67]). In isogenic heterozygous R1441C iPSCs, we detected a modest increase in basal pT73 Rab10 immunostaining relative to WT cells, that was reduced by acute MLi-2 treatment (Supplementary Fig. 2D, E). In addition, treatment with the lysosomotropic agent LLOMe, reported to induce recruitment and activation of LRRK2 at damaged lysosomes [28, 68], induced strong phosphorylation of Rab12 and a mild increase in phosphorylated Rab10 levels, in WT iPSCs (Supplementary Fig. 2F), consistent with endogenous LRRK2-dependent signaling. NGN2-induced glutamatergic neurons generated from this isogenic LRRK2 series validated these phenotypes with an increase in the labile iron pool across R1441C, Y1699C and G2019S heterozygous mutant iNs, compared to WT controls (Fig. 1L), supporting a convergent effect of pathogenic LRRK2 mutations on iron-related phenotypes in neurons.

### LRRK2 mutations cause kinase-dependent accumulation of lysosomal iron in neurons and astrocytes

The FerroOrange iron probe is reported to detect free ferrous iron in the cytosol as well as in different organelles including ER, mitochondria and endolysosomes [69–71] (Supplementary Fig. 3A). Thus, the data in Fig. 1 do not differentiate between cytosol and specific subcellular compartments. Given the role of lysosomes as important sites for iron mobilization regulating intracellular iron availability [72–74], and the well-established role of LRRK2 in lysosome-related pathways reported by us and others [28, 29, 32, 68, 75, 76], we focused on evaluating lysosomal iron in LRRK2 mutant cells.

**Fig. 2:**
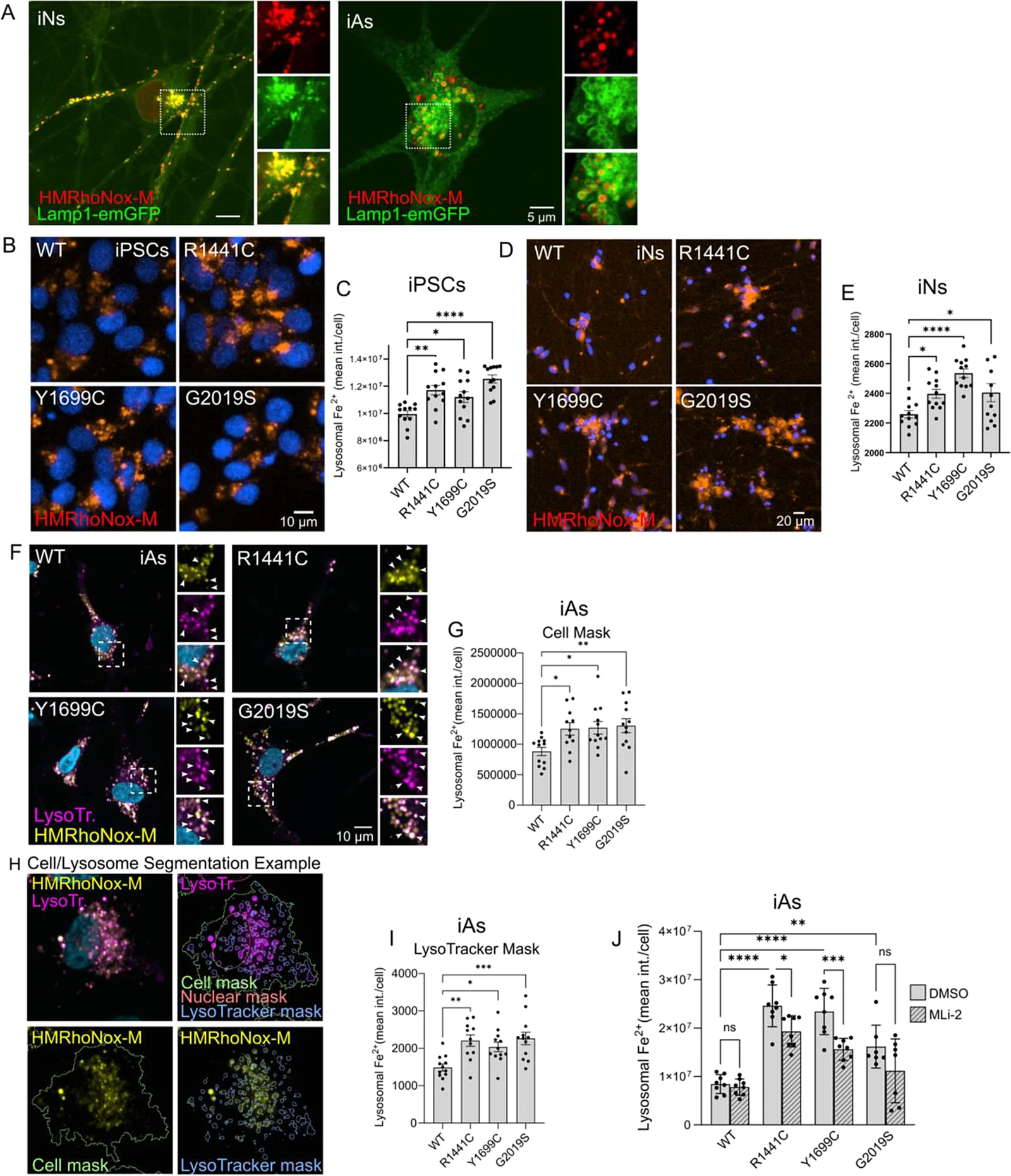
LRRK2 mutations increase lysosomal iron in isogenic neurons and astrocytes. (**A**). Representative images of lysosomal iron (HMRhoNox-M) and Lamp1-emGFP by super-resolution confocal microscopy in iNs and iAs. Lysosomal iron imaging and quantitation in heterozygous LRRK2 mutant isogenic iPSCs (**B, C**), isogenic iNs (**D, E**), and isogenic iAs (**F, G**). Quantification was performed using a whole-cell mask to capture total cellular HMRhoNox-M signal (one-way ANOVA, Dunnett’s post-hoc, *N* = 8 biological replicates, (> 800 cells per N), *p < 0.05, **p < 0.01, ***p < 0.001, ****p < 0.0001). (**H**) Representative images illustrating LysoTracker-based segmentation of acidic lysosomal compartments and corresponding detection of lysosomal iron using HMRhoNox-M. Cell, nuclear, and LysoTracker masks used for analysis are shown. (**I**) Quantification of HMRhoNox-M signal specifically within LysoTracker-segmented lysosomes in isogenic iAs, demonstrating genotype-dependent increases in lysosomal iron that recapitulate those observed using whole-cell mask-based quantification. Data are shown as mean ± SEM (one-way ANOVA with Dunnett’s post hoc test). (**J**) Lysosomal iron quantitation in iAs following treatment with MLi-2 in culture (100 nM, 7 days) (two-way ANOVA, Tukey’s post-hoc; *N* = 8 biological replicates (> 800 cells per N), Genotype ****p = 0.0001, Treatment ****p < 0.0001, Interaction *p* = 0.0844)

We thus employed a reported lysosomal specific ferrous iron (Fe^2+^) probe (HMRhoNox-M) [77] to assess relative lysosomal Fe^2+^ levels across iPSCs, NGN2-induced neurons (iNs) and induced-astrocytes (iAs) [59] using high-content imaging and super-resolution microscopy (Fig. 2). iAs were generated by viral delivery of doxycycline-inducible expression of SOX9 and NFIB [59]. The expression of astrocytic markers and LRRK2 was confirmed at ~ 21 days post-induction in vitro (Supplementary Fig. 2). We first validated the lysosomal specificity of HMRhoNox-M by comparing its staining pattern to Lamp1-emGFP localization (Fig. 2A). In both iPSC-derived human neurons and astrocytes (Fig. 2A), HMRhoNox-M signal was enriched within Lamp1-emGFP positive structures, supporting its preferential localization to lysosomes. Quantification of HMRhoNox-M signal in iPSCs carrying heterozygous LRRK2 mutations revealed an increase in R1441C, Y1699C and G2019S LRRK2 mutant lines compared to isogenic WT controls (Fig. 2B, C). The LRRK2 mutation-driven increase in lysosomal iron as detected by HMRhoNox-M was likewise observed in differentiated iNs (Fig. 2D, E) and astrocytes (Fig. 2F, G) generated from these parental isogenic iPSCs.

Notably, not all HMRhoNox-M-positive puncta overlapped with Lamp1-emGFP-labeled structures, particularly in astrocytes (Fig. 2A), which could reflect incomplete labeling of acidic lysosomal compartments by the Lamp1 fusion protein [78]. To address this possibility directly, we performed complementary analyses using LysoTracker, which labels acidic lysosomes independently of exogenous Lamp1 expression (Fig. 2F; Supplementary Fig. 3). Using this approach, HMRhoNox-M showed strong colocalization with LysoTracker-positive compartments (Supplementary Fig. 3A). Quantification of HMRhoNox-M selectively within LysoTracker-segmented lysosomes (Fig. 2H, I) recapitulated the genotype-dependent increases observed using a whole-cell mask-based analysis (Fig. 2G, H). Finally, to exclude the possibility that differences in lysosomal Fe^2+^ levels reflected changes in lysosome abundance or morphology rather than iron content per organelle, we quantified lysosome number and size across the isogenic lines. No significant differences in these parameters were observed in heterozygous LRRK2 mutant iAs (Supplementary Fig. 3B-E), indicating that elevated HMRhoNox-M signal reflects increased lysosomal iron load rather than altered lysosomal biogenesis or expansion.

Given that LRRK2 can localize to lysosomal membranes and that lysosomal position has been reported to correlate with LRRK2 kinase activity toward Rab GTPases [79], we next examined whether iron accumulation exhibited spatial heterogeneity within the lysosomal network. Quantitative analysis revealed that the increase in lysosomal iron content in R1441C iAs was more pronounced in the perinuclear compared to the peripheral lysosomes and that this increased iron load was preferentially associated with smaller lysosomes (< 3 µm^3^) (Supplementary Fig. 3F, G, H). These data are consistent with perinuclear lysosomes being sites of active LRRK2 signaling [79], as demonstrated by work from us and others showing LRRK2-dependent effects on lysosomal function and proteolytic activity [26, 56, 80, 81].

To determine whether the increase in lysosomal iron observed in LRRK2 mutant iAs is dependent on LRRK2 kinase activity, iAs were treated with MLi-2 for 7 days prior to analysis (Fig. 2J). LRRK2 inhibition reduced lysosomal iron levels in all mutant lines but not WT, with partial rescue in the R1441C and Y1699C lines and complete rescue in the G2019S line (Fig. 2J). Effective inhibition of endogenous LRRK2 kinase activity was validated in parallel iPSC cultures by assessing Rab10 phosphorylation at Threonine 73, which showed a modest increase in R1441C iPSCs and was reduced by acute MLi-2 (2 h) treatment (Supplementary Fig. 2D, E). To assess the selectivity of MLi-2 on LRRK2-mediated effects on iron homeostasis, LRRK2 KO iPSCs were treated with vehicle or MLi-2 for either acute (2 h) or prolonged (7 day) durations and cytosolic and lysosomal iron levels were assessed. MLi-2 had no effect on iron levels in LRRK2 KO cells (Supplementary Fig. 4A-D), indicating that MLi-2 as employed here does not possess off-target effects with respect to iron homeostasis. Together, these results provide evidence that lysosomal iron levels are increased across multiple pathogenic LRRK2 mutations, and that this phenotype is partially sensitive to LRRK2 kinase inhibition with MLi-2.

### Ferritin levels are dysregulated in isogenic LRRK2 mutant iPSCs

**Fig. 3:**
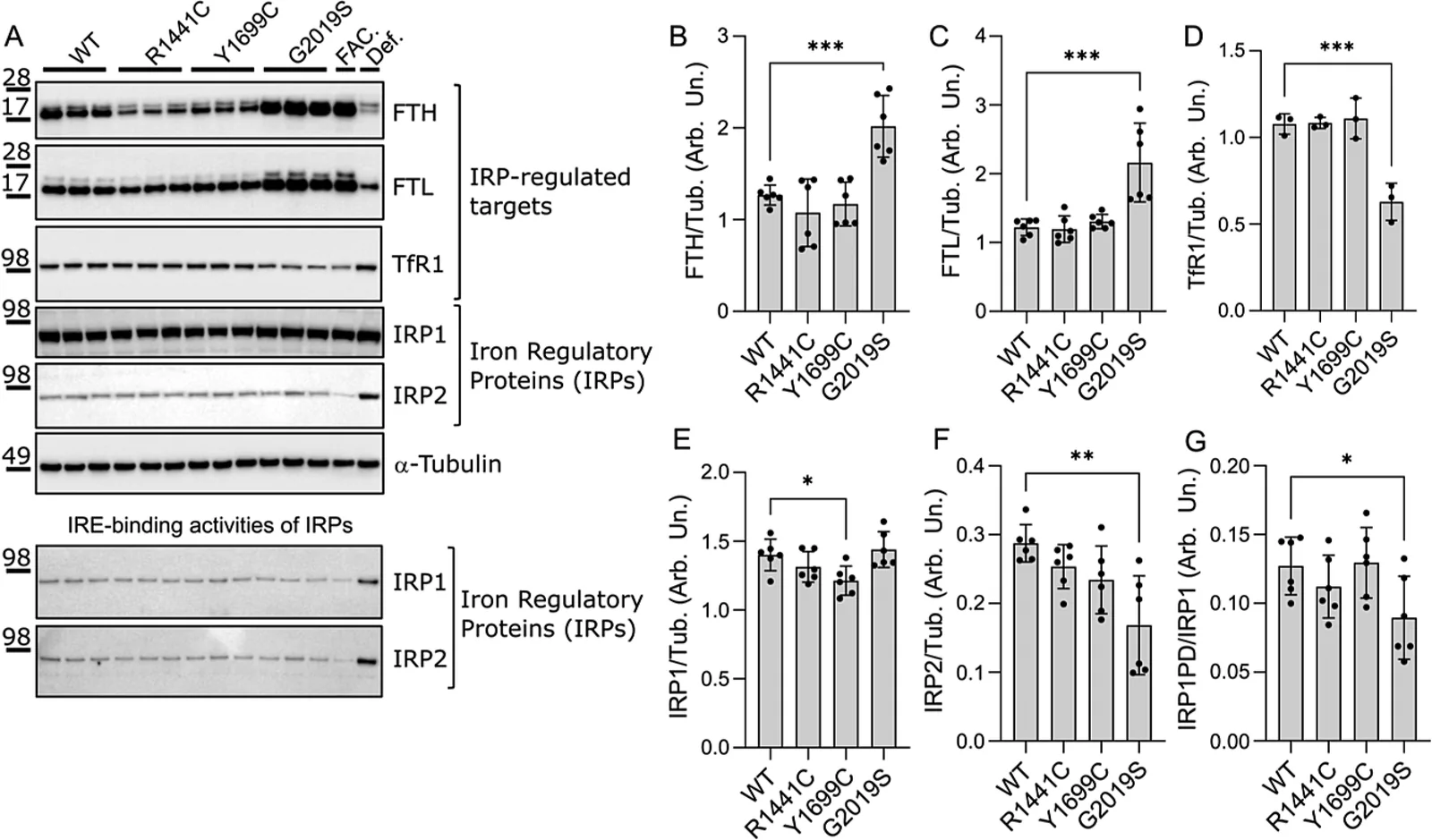
Ferritin heavy chain is dysregulated in isogenic LRRK2 mutant iPSCs. (**A-F**) Western blot analysis of IRPs (IRP1, IRP2) and IRP-regulated proteins (FTH1, FTL, TfR1) in isogenic LRRK2 mutant iPSCs. (A bottom panel, G) Western blot of IRE-bound IRP1 in isogenic LRRK2 mutant iPSCs and quantitation of IRE-binding (IRP1PD: IRP1 pull-down). (one-way ANOVA, Dunnett’s post-hoc, *N* = 6 biological replicates, *p < 0.05, **p < 0.01, ***p < 0.001)

Altered cytosolic and lysosomal iron demonstrate an impairment in how cells regulate their iron levels and distribution, so we further evaluated expression of key proteins involved in these processes. Iron uptake, transport, and storage are finely regulated at the post-transcriptional level by the iron regulatory protein (IRP) and iron-responsive element (IRE) signaling pathways [82]. IRP1 and IRP2 are RNA-binding proteins that interact with IREs located within the untranslated regions of specific mRNAs, to modulate iron homeostasis. These interactions repress ferritin (both FTH1 and FTL) and FPN mRNA translation while stabilizing TfR mRNA [83–85]. In proliferating iPSCs cultured without iron supplements, we observed an increase in total FTH1 and FTL levels in G2019S LRRK2 cells compared to isogenic WT controls, while R1441C LRRK2 iPSCs showed a trend of lower FTH1 (Fig. 3A, B, C). Consistent with an IRP-mediated loss of translational repression of ferritin heavy and light chains, G2019S iPSCs also had lower basal TfR1 levels as compared to WT (Fig. 3A, D). We therefore directly measured IRP1 and IRP2 levels and activity in these LRRK2 mutants. We observed a trend of downregulation of IRP1 in the ROC-COR domain mutants that reached significance in Y1699C iPSCs, supporting impairment in IRP1 signaling (Fig. 3A, E). Separately, the G2019S iPSCs showed lower IRP2 expression levels with the ROC-COR domain mutants showing a trend toward decrease (Fig. 3F). To evaluate IRE-binding activities of IRP1 and IRP2, we used a biotinylated-IRE probe to separate IRE-binding IRPs from cell lysates and analyzed by immunoblotting, as before [63]. The IRE-binding activity of IRP1 was lower in G2019S iPSCs compared to the isogenic WT (Fig. 3G), as were IRP2 protein levels and IRE-binding activity (Fig. 3A).

IRP1 activity is regulated by intracellular iron levels but can also be influenced by Fe-S cluster biogenesis as it requires a 4Fe-4S cluster to function as a cytosolic aconitase [83, 86]. Loss of the Fe-S cluster, under iron deficient conditions, shifts IRP1 to its IRE-binding form. In contrast, IRP2, does not coordinate an Fe-S cluster, and cellular iron availability is directly reflected by IRP2 protein levels, which increase when iron is scarce and decrease through degradation when iron is sufficient. To evaluate a potential effect of iron dyshomeostasis on mitochondrial function, we assayed levels of mitochondrial Fe-S-containing subunits (Supplementary Fig. 5), noting no differences in basal levels of UQCRFS1 (Fe-S subunit of complex III), and SDHB (Fe-S subunit of complex II). Overall, these data indicate mutation-associated changes in ferritin/IRP-related pathways, with the clearest effects observed in G2019S cells, but do not necessarily link all LRRK2 mutations to ferritin regulation.

#### LRRK2-Rab8a signaling contributes to cellular iron homeostasis

**Fig. 4:**
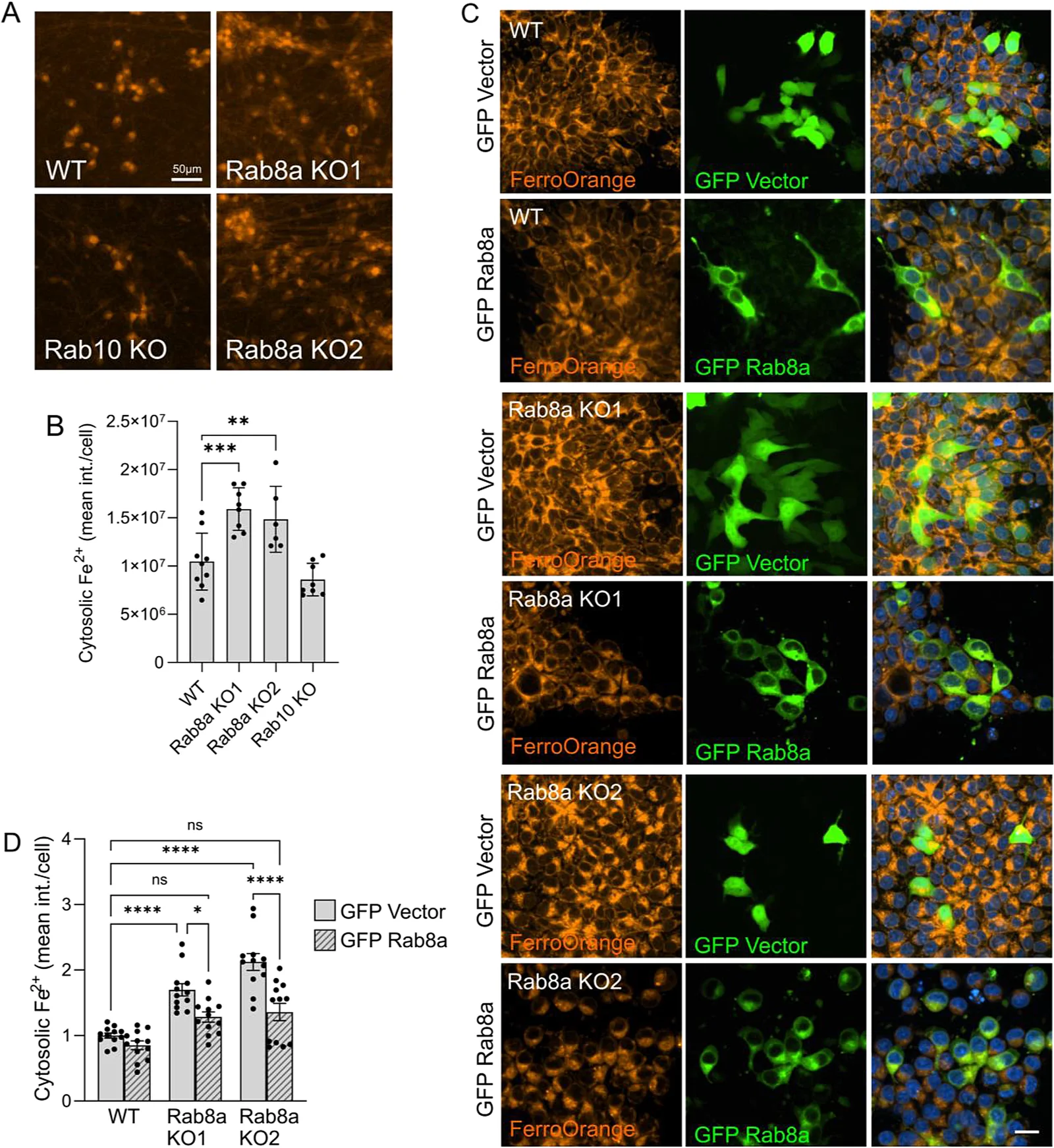
Knockout of the LRRK2 substrate Rab8a dysregulates cellular iron. (**A, B**) Imaging of cytosolic free Fe^2+^ iron and quantitation of iron levels in isogenic WT, Rab8a KO and Rab10 KO iNs by high-content imaging (one-way ANOVA, Dunnett’s post-hoc, *N* > 6 biological replicates, *p < 0.05, **p < 0.01, ***p < 0.001, ****p < 0.0001). (**C, D**) Representative images and quantitation of free cytosolic iron staining in WT and Rab8a KO iPSCs expressing either GFP control vector or GFP-Rab8a (two-way ANOVA, Tukey post-hoc; *N* = 12 biological replicates (> 800 cells per N), Genotype ****p < 0.0001, cDNA ****p < 0.0001, Interaction **p = 0.0073)

Phosphorylation of Rab GTPases by LRRK2 alters their interactions with effectors and regulators, thereby changing trafficking behavior and downstream function [32, 36, 56, 87]. We previously showed that LRRK2-driven phosphorylation of Rab8a impairs its function in TfR trafficking, linking LRRK2 to iron homeostasis [32]. Furthermore, we demonstrated that Rab8a and Rab10 knockouts (KO) produce distinct phenotypes in lysosomal integrity and PD-pathology related proteostasis [56]. To test whether genetic inactivation of either of these Rab proteins is sufficient to drive dysregulation of cellular iron levels, we used CRISPR/Cas9 gene editing to generate Rab8a KO and Rab10 KO iPSCs [56] and HEK293 cells (Supplementary Fig. 6), and assayed cellular iron load alongside matched isogenic controls. Using the FerroOrange probe to quantify labile cytosolic Fe^2+^ iron, we found that Rab8a KO but not Rab10 KO iNs showed an increase in free iron compared to isogenic WT controls, phenocopying the increase observed in mutant LRRK2 cells (Fig. 4A, B). To determine whether Rab8a deficiency is causative for this phenotype, we next tested whether re-expression of Rab8a could restore iron homeostasis. Expression of exogenous GFP-Rab8a in Rab8a KO iPSCs significantly reduced FerroOrange signal, normalizing labile cytosolic Fe^2+^ iron levels toward those observed in WT cells (Fig. 4C, D). These findings show that Rab8a loss is associated with increased labile cytosolic Fe^2+^ iron levels and phenocopies a subset of iron-related phenotypes observed in LRRK2 mutant cells. Notably, this increase in labile iron was specific to Rab8a loss, as Rab10 deficiency resulted in a divergent phenotype in iPSCs and HEK293 cells, supporting non-redundant roles for LRRK2-regulated Rab GTPases in iron homeostasis (Supplementary Fig. 7).

To further assess how Rab8a and Rab10 deficiency influence cellular iron handling, we examined iron-related protein expression in Rab8a and Rab10 KO HEK293 cells under basal conditions and following overnight exposure to 150 µM ferric ammonium citrate (FAC). Rab8a KO cells exhibited elevated basal FTH1 levels compared to WT and Rab10 KO cells but showed a blunted FTH1 induction following FAC treatment (Supplementary Fig. 7). In contrast, Rab10 KO cells displayed a robust increase in FTH1 upon iron overload. Basal TfR1 levels were increased in Rab10 KO cells, while both Rab KO lines exhibited a greater reduction in TfR1 expression following FAC treatment relative to WT. Consistent with these protein changes, Rab8a KO cells accumulated higher levels of labile iron under iron overload conditions, which was reversed by iron chelation. Together, these data further support Rab8a loss as a contributor to cellular iron accumulation; while highlighting that not all LRRK2-regulated Rab GTPases exert equivalent effects on iron-handling pathways.

**Fig. 5:**
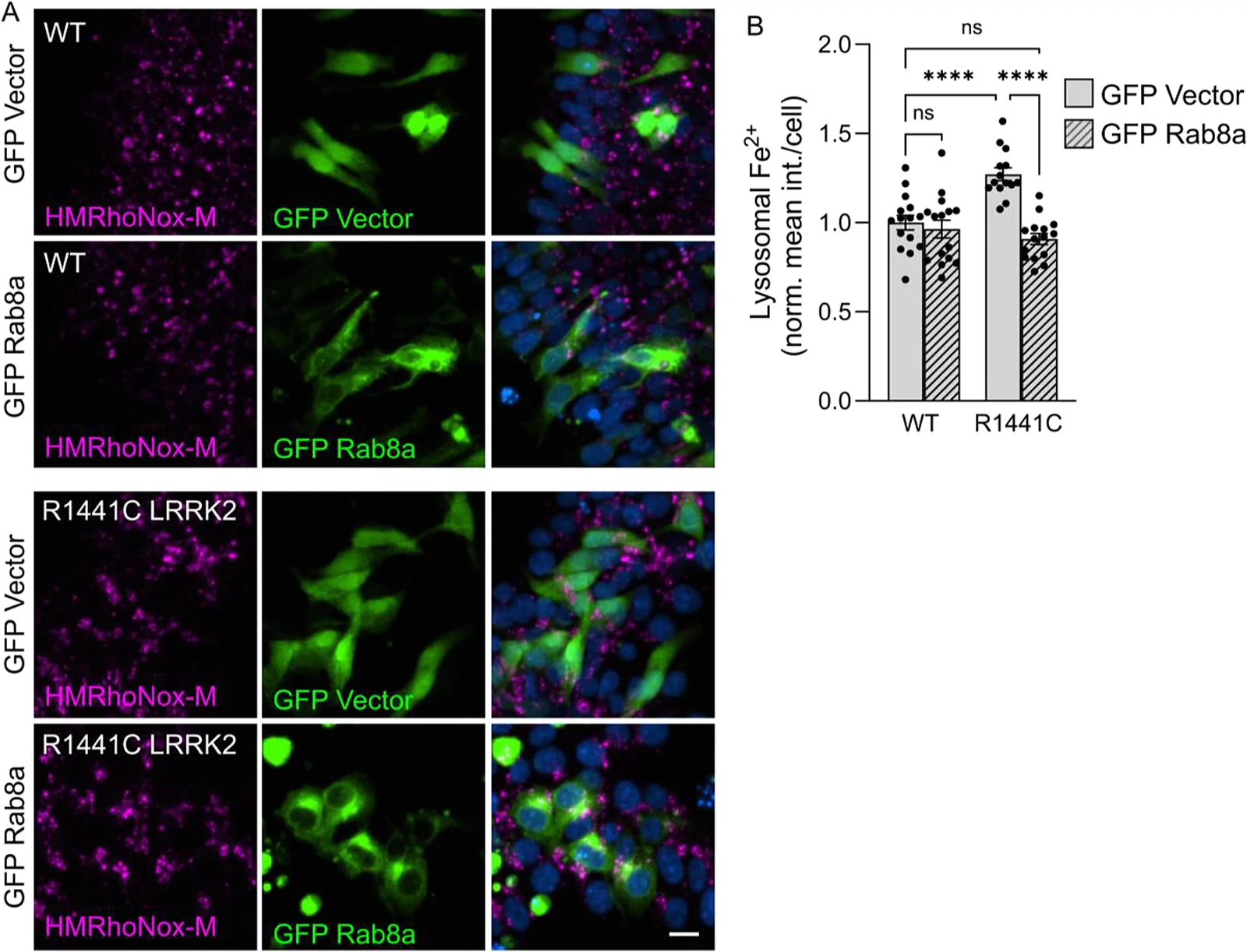
Exogenous Rab8a expression normalizes lysosomal iron levels in R1441C LRRK2 iPSCs. (**A, B**) Representative images and quantitation of lysosomal iron in WT and R1441C LRRK2 iPSCs expressing either GFP control vector or GFP-Rab8a. (two-way ANOVA, Tukey post-hoc; *N* = 15 biological replicates (> 800 cells per N), Genotype *p* = 0.0096, cDNA ****p < 0.0001, Interaction ***p = 0.0002)

The LRRK2-dependent increase in lysosomal Fe^2+^ iron was the most consistent phenotype across all pathogenic mutations and cell types analyzed. Since LRRK2 mutations alter Rab substrate effector interactions and function, we examined whether exogenous Rab8a over-expression could overcome this effect in R1441C LRRK2 mutant iPSCs. WT and R1441C iPSCs were transiently transfected to express either GFP or GFP-Rab8a and lysosomal iron was quantified in positively transfected cells. GFP-Rab8a expression in WT iPSCs resulted in a minor reduction in iron that did not reach statistically significant deviation from the GFP control. R1441C iPSCs transfected with GFP showed increased lysosomal iron compared to WT cells, as expected. However, expression of GFP-Rab8a in R1441C iPSCs significantly reduced lysosomal iron levels to those comparable to WT (Fig. 5A-B). Collectively, these data identify Rab8a as an important contributor to LRRK2-associated iron dyshomeostasis and demonstrate that exogenous Rab8a expression can normalize iron phenotypes associated with LRRK2 mutations.

### Iron chelation and LRRK2 inhibition protect against LRRK2-driven oxidative stress and lipid peroxidation

**Fig. 6:**
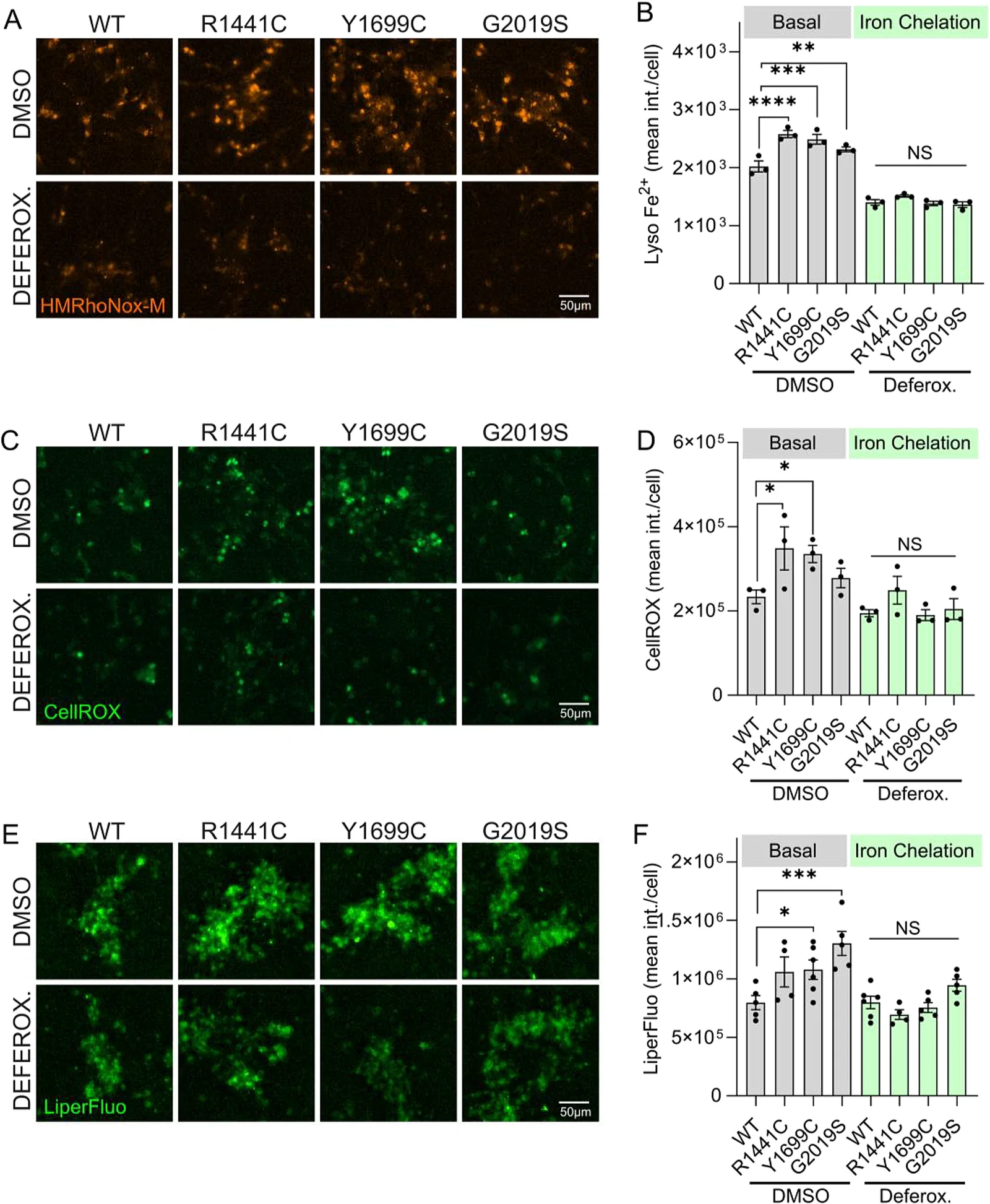
Iron chelation reduces LRRK2-associated ROS accumulation and lipid peroxidation. (**A, B**) High-content imaging and quantitation of lysosomal iron content (HMRhoNox-M) in iNs carrying heterozygous LRRK2 mutations versus isogenic WT controls, following treatment with 100 µM deferoxamine (16 h) in normal media. (**C, D**) High-content imaging and quantitation of CellROX dye in LRRK2 mutant iNs. (E, F) Lipid peroxidation imaging (Liperfluo) and quantitation in iNs. (HMRhoNox-M: two-way ANOVA, Tukey post-hoc; *N* = 3 biological replicates (> 800 cells per N), Genotype ***p = 0.0003, Treatment ****p < 0.0001, Interaction **p = 0.0034; CellROX: two-way ANOVA, Tukey post-hoc; *N* = 3 biological replicates (> 800 cells per N), Genotype *p = 0.02, Treatment ***p = 0.0002, Interaction *p* = 0.3923; Liperfluo: two-way ANOVA, Tukey post-hoc; *N* = 3 biological replicates (> 800 cells per N), Genotype ***p = 0.0009, Treatment ****p < 0.0001, Interaction *p = 0.0497)

Ferroptosis, the process of iron-mediated lipid peroxidation and cell death, is an emerging pathogenetic mechanism of interest in the neurodegeneration in PD [20, 88–90]. Iron catalyzes the conversion of hydrogen peroxide to more reactive free radicals via the Fenton reaction thereby promoting oxidative injury. Ferroptosis in neurodegeneration involves the simultaneous accumulation of brain iron and lipid peroxidation, which trigger a cascade of pathologic events including inflammation, myelin sheath degeneration, glial dysregulation and cell death [91]. LRRK2 mutations have been linked to increased ROS [50, 51] and ferroptosis signaling [92, 93] in different model systems but the mechanism by which LRRK2 signaling leads to increased cell stress remains largely unclear. Here, we examined whether dysregulated lysosomal iron in LRRK2 mutant neurons contributes to oxidative stress and downstream lipid peroxidation. Compared to isogenic WT controls, all three LRRK2 mutations led to increased lysosomal iron in differentiated iNs (Fig. 6A, B). As expected, the iron chelator deferoxamine (100 µM; 16 h) significantly reduced lysosomal iron levels below control in all cells (Fig. 6A, B). Assessment of cytoplasmic ROS using CellROX revealed a genotype-dependent increase in oxidative stress in heterozygous LRRK2 mutant neurons, which was reduced by deferoxamine treatment, consistent with its ability to chelate iron in the media (Fig. 6C, D). We similarly assessed lipid peroxidation with Liperfluo, which produces a prominent punctate, vesicular pattern by confocal microscopy (Supplementary Fig. 8A) [94], and used erastin, a ferroptosis inducer as a positive control (Supplementary Fig. 8B, C) [95–98]. All three pathogenic mutations in LRRK2 significantly increased lipid peroxidation levels in iNs, which was likewise attenuated by iron chelation with deferoxamine (Fig. 6E, F). Similarly, at basal conditions in proliferating cells, we detected an increase in lipid peroxidation in R1441C and G2019S iPSCs compared to isogenic WT controls (Supplementary Fig. 8B, C). These data are in line with recent work that places LRRK2 in a ferroptosis pathway and highlight lipid peroxidation as a relevant mechanism in LRRK2 signaling [92, 93]. Together, these findings demonstrate that pathogenic LRRK2 mutations promote iron-dependent oxidative stress and lipid peroxidation in neurons, consistent with a pro-ferroptosis phenotype.

**Fig. 7:**
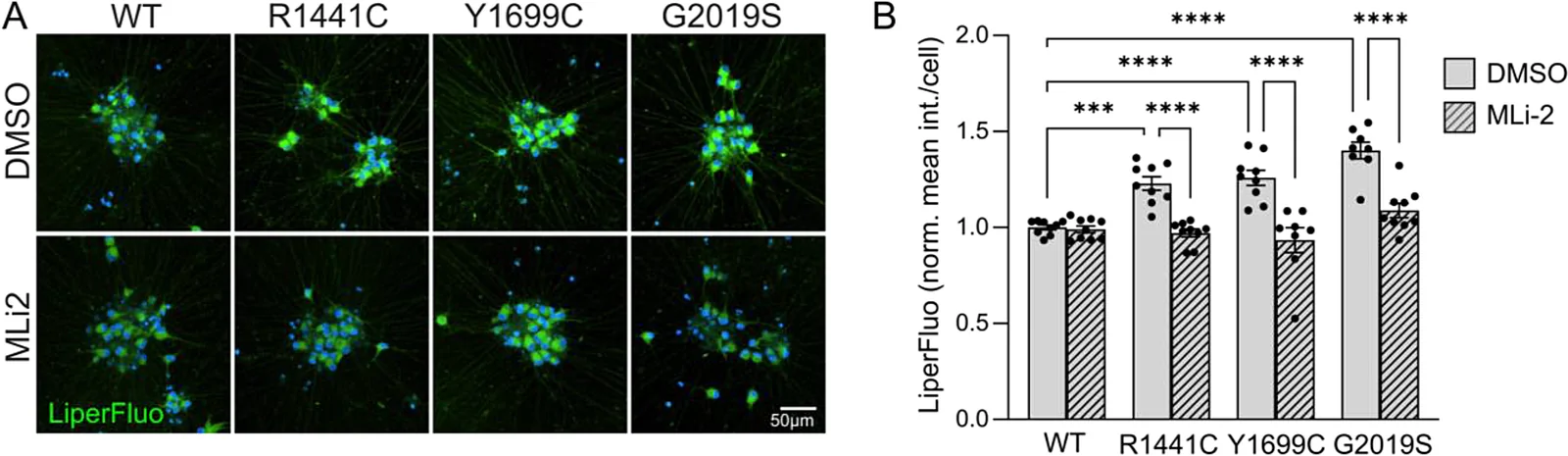
Selective kinase inhibition reduces lipid peroxidation in LRRK2-mutant neurons. (**A, B**) Representative images and quantitation of lipid peroxidation detected by Liperfluo in mature iNs expressing WT or isogenic LRRK2 variants following treatment with vehicle or MLi-2 (100 nM, 7 days) (two-way ANOVA, Tukey post-hoc; *N* = 9 biological replicates (> 800 cells per N), Genotype *p* < 0.0001, Treatment *p* < 0.0001, Interaction *p* < 0.0001)

To determine whether LRRK2 kinase activity contributes directly to lipid peroxidation, we next assessed the effect of pharmacological LRRK2 inhibition using MLi-2. To assess the selectivity of this inhibitor, LRRK2 KO iPSCs were treated with vehicle or MLi-2 (100 nM) for 7 days, with fresh inhibitor applied every 48 h. No differences in basal Liperfluo signal were observed, confirming the selectivity of MLi-2 (Supplementary Fig. 8D, E). We then examined the effect of selective LRRK2 kinase inhibition on lipid peroxidation in LRRK2 mutant iNs. Treatment of mature iNs with MLi-2 significantly reduced Liperfluo signal in R1441C, Y1699C, and G2019S neurons, reducing lipid peroxidation levels toward those observed in WT controls (Fig. 7A, B). These data indicate that lipid peroxidation is increased in LRRK2 mutant neurons in a kinase-dependent manner.

#### LRRK2 kinase-dependent iron dysregulation in human DA neurons

**Fig. 8:**
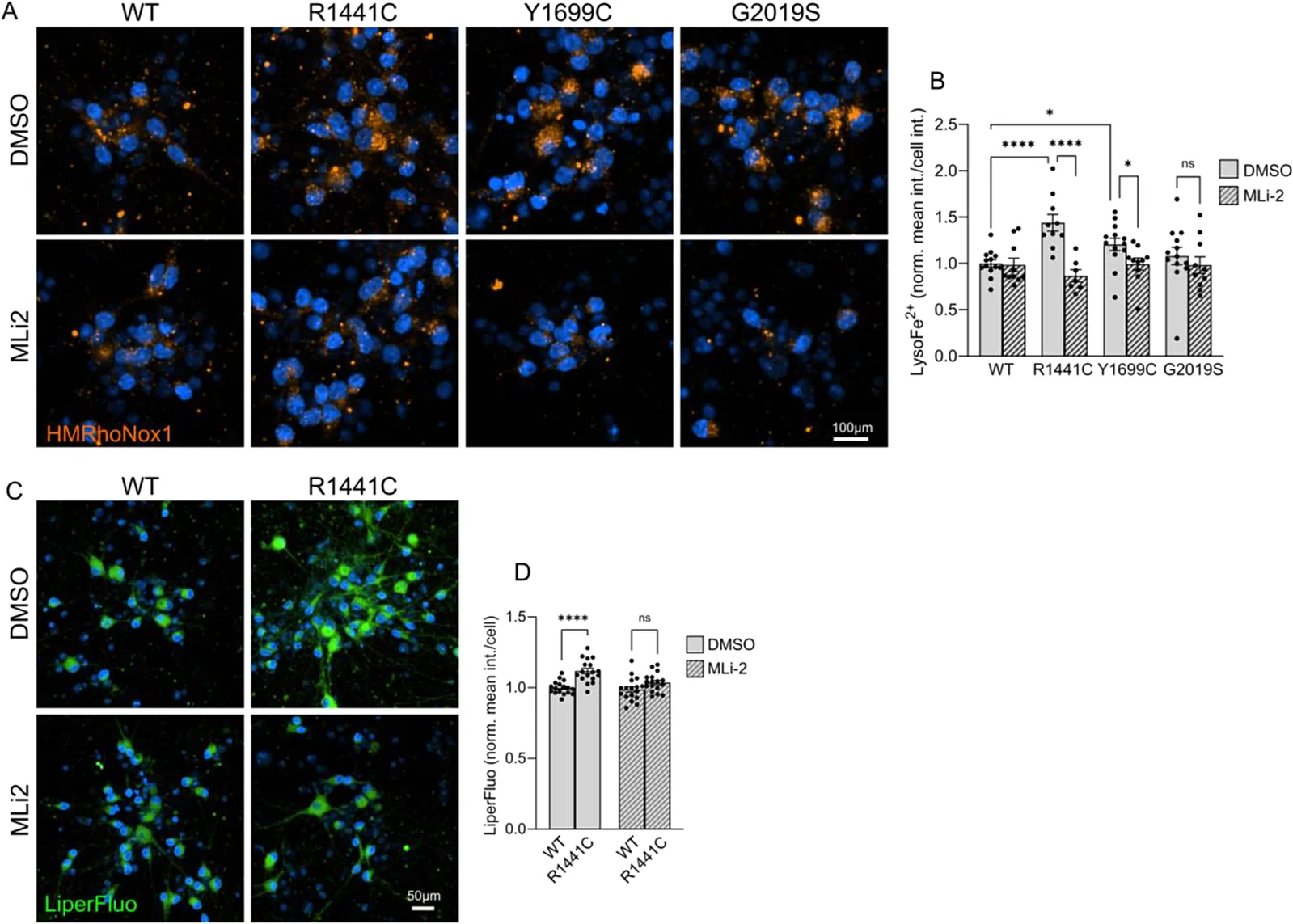
LRRK2 kinase inhibition modulates iron and lipid peroxidation in human dopaminergic neurons. (**A, B**) Representative images and quantitation of lysosomal iron (HMRhoNox-M) in mature human iPSC-derived DA neurons of the indicated genotypes following treatment with vehicle (DMSO) or MLi-2 (100 nM, 7 days) (two-way ANOVA, Tukey post-hoc; *N* > 10 biological replicates (> 800 cells per N), Genotype *p* = 0.1953, Treatment *p* < 0.0001, Interaction *p* = 0.0061). (**C, D**) Representative images and quantitation of lipid peroxidation (Liperfluo) in WT and heterozygous R1441C DA neurons following treatment with DMSO or MLi-2. (two-way ANOVA, Tukey post-hoc; *N* = 15 biological replicates (> 800 cells per N), Genotype *p* < 0.0001, Treatment *p* = 0.0148, Interaction *p* = 0.1211)

Given the selective vulnerability of DA neurons in PD, we next examined whether LRRK2 mutations dysregulate iron homeostasis and oxidative stress in human iPSC-derived DAs. DA neurons were generated from isogenic WT and LRRK2 mutant iPSCs using established protocols [61, 62] and TH positivity was confirmed by ICC (Supplementary Fig. 9A). Mature DAs carrying LRRK2 mutations were treated with the LRRK2 kinase inhibitor MLi-2 for 7 days prior to analysis. In DA neurons, pairwise comparisons indicated increased lysosomal iron in R1441C and Y1699C cultures relative to WT, despite heterogeneity across genotypes in the overall model, with these effects rescued by MLi-2 treatment (Fig. 8A, B). Subsequent lipid peroxidation analyses were focused on R1441C DA neurons, as this mutation showed robust Rab8a phosphorylation [32] and the most pronounced changes across multiple iron-related readouts examined here. R1441C DA neurons exhibited significantly increased lipid peroxidation compared to WT controls (Fig. 8C, D). Importantly, the LRRK2 mutation-driven increase in lipid peroxidation was significantly reduced by selective LRRK2 kinase inhibition in DA neurons, with no effect in WT DA neurons (Fig. 8C, D). To verify the selectivity of MLi-2 for LRRK2 kinase activity in DA neurons, as previously established in iPSCs (Supplementary Fig. 8), LRRK2 KO DA neurons were treated with vehicle or 100 nM MLi-2 for 7 days. MLi-2 had no effect on Liperfluo signal in the LRRK2 KO DA neurons, confirming its specificity for this measure in these neurons (Supplementary Fig. 9B, C). Together, these data demonstrate that pathogenic LRRK2 mutations promote lysosomal iron accumulation and lipid peroxidation in human DA neurons, with both phenotypes dependent on a pathogenic increase in LRRK2 kinase activity.

## Discussion

The pathological deposition of iron in the PD brain has been widely documented for over three decades, with early histochemical studies identifying iron accumulation in the substantia nigra of post-mortem PD tissue [99, 100], and subsequent MRI and spectroscopic studies confirming region-specific iron overload in vivo [9, 101, 102]. However, the mechanisms by which genetic risk factors may contribute to intracellular iron mismanagement have remained poorly defined. In this study we show that PD-linked LRRK2 mutations are associated with iron mishandling in human iPSC-derived neurons and astrocytes, with a convergent increase in lysosomal ferrous iron. This iron accumulation was partially sensitive to LRRK2 kinase inhibition, implicating pathogenic LRRK2 signaling in the misregulation of lysosomal iron handling. Our data indicate Rab8a as an important contributor to this phenotype, and link altered iron handling to oxidative stress and lipid peroxidation in PD-relevant cell models. Although experiments in undifferentiated iPSCs provide supportive and genetically tractable evidence for LRRK2/Rab8a involvement, the central conclusions of this study are based on convergent findings in differentiated iPSC-derived glutamatergic neurons, dopaminergic neurons, and astrocytes.

Most pathogenic LRRK2 mutations cluster within the kinase and the Roc-COR tandem domains of the protein, and all are thought to augment the kinase activity of LRRK2 towards a subset of Rab GTPases [34]. Rab GTPases control many aspects of intracellular vesicle trafficking by acting as regulatable switches that recruit effector molecules to distinct intracellular membranes [36, 103]. We and others have shown that phosphorylation of Rab GTPases by mutant LRRK2, including Rab8a and Rab10, interferes with their function and alters endolysosomal dynamics [26, 28, 32, 34, 35, 104–106]. Furthermore, it has been reported that recruitment of LRRK2 to damaged lysosomes, along with Rab8a and Rab10, affects lysosomal integrity [28, 32, 68]. Studies have shown opposing effects of Rab8a and Rab10 in ciliogenesis [67] and we recently reported divergent lysosomal phenotypes between Rab8a KO and Rab10 KO cells [56]. Here, we extend this work by demonstrating that loss of Rab8a but not Rab10 partially mimics aspects of iron impairment observed in LRRK2 mutant cells, while exogenous Rab8a expression can rescue lysosomal iron accumulation in R1441C LRRK2 mutant cells. Together, these findings support a Rab8a-sensitive arm of LRRK2 iron-related phenotypes, potentially through its role in transferrin receptor trafficking [32, 40], while also indicating that additional mutation-specific consequences of pathogenic LRRK2 likely contribute. Prior work indicates that endogenous LRRK2-dependent Rab phosphorylation can occur at low overall stoichiometry, while still producing functional effects through localized phospho-Rab pools and altered effector interactions. Thus, pathogenic LRRK2 may act on a spatially or functionally restricted pool of Rab8a at lysosomal or recycling compartments, where even low stoichiometry phosphorylation could meaningfully affect iron trafficking. We previously reported that loss of Rab8a perturbs α-synuclein proteostasis in human neurons [56]. Given prior evidence that iron can post-transcriptionally modulate α-synuclein protein synthesis and directly influence its conformation and fibrilization [107–109], it will be important in future studies to determine whether iron dyshomeostasis contributes to abnormal α-synuclein handling downstream of LRRK2-Rab8a dysfunction.

Lysosomes are key hubs for iron storage and availability [73, 74]. Endolysosomal acidification is required for iron release and trafficking, and its disruption has been linked to cellular iron deficiency, mitochondrial dysfunction, and inflammation [74, 110, 111]. Here, using a lysosomal-specific iron probe we found that LRRK2 mutations are associated with increased lysosomal ferrous iron content in neurons and astrocytes. These data, together with previous reports linking LRRK2 to lysosomal function [28, 32, 68], and intralumenal pH [26, 81], support altered lysosomal iron trafficking as a plausible consequence of pathogenic LRRK2 signaling. We observed greater ferrous iron accumulation in perinuclear lysosomes in LRRK2 mutant astrocytes, which is notable given reported differences in luminal pH and protease activity across lysosomal subpopulations [80] and evidence that perinuclear lysosomes show enhanced LRRK2-dependent Rab signaling [79]. Consistent with prior reports of altered lysosomal acidification in LRRK2 mutant models [26, 31, 81, 112, 113], our findings suggest that pathogenic LRRK2 signaling may modulate lysosomal iron mobilization by altering lysosomal homeostasis.

Iron-induced oxidative stress is a well-documented contributor to neurodegeneration, and our results support a role for LRRK2 mutations in exacerbating this process in DA neurons. Iron-induced cell death, termed ferroptosis, has emerged as a key mechanism contributing to oxidative damage and neuronal loss in PD models [20, 88–90]. The convergence of aberrant brain iron and lipid peroxidation initiates downstream pathology marked by inflammation, glial dysfunction, demyelination, and ultimately neuronal death [91]. Here, we observed increased lipid peroxidation and ROS levels in mutant LRRK2 DA neurons and glutamatergic iNs, which were reversed by iron chelation and LRRK2 inhibition. LRRK2 has been linked to ROS accumulation in different models [50–52], while a recent study reported an effect of LRRK2 mutations in NOX2 activity that in turn has been linked to ferroptosis [51, 114, 115]. Furthermore, studies have linked G2019S LRRK2 with heightened oxidative stress vulnerability in human iPSC-derived DA neurons [116], while more recently, in vivo data suggested age-dependent oxidative stress and mitochondrial dysfunction in the SNc of R1441G LRRK2 BAC transgenic mice [117]. These data are consistent with mitochondrial abnormalities and dopaminergic alterations reported in G2019S knockin mice [118]. It is plausible that dysregulation of the labile and lysosomal iron pools by LRRK2 mutations contributes to the reported effects of LRRK2 on cellular ROS. The lysosomal membrane is exposed to redox-active iron and is an initial target of intracellular oxidant damage, with studies reporting a protective role for intra-lysosomal iron chelation against ROS damage [119]. What remains to be established is whether dysregulation of lysosomal Fe^2+^ levels by mutant LRRK2 solely drives cellular ROS damage, or whether aberrant transport of endolysosomal iron to the labile or mitochondrial iron pools also contribute pathologic effects. The finding that deferoxamine attenuated both ROS and lysosomal iron content further strengthens the connection between lysosomal iron mismanagement and oxidative stress in PD-relevant cell types. Ferroptosis inhibitors have shown promise in mitigating neurodegeneration in cellular and in vivo preclinical models [20, 91, 120]. Our data point to lysosomal iron dysregulation as one tractable node within the broader oxidative stress pathway engaged by pathogenic LRRK2.

Cellular iron homeostasis is tightly regulated at the post-transcriptional level by IRPs, which bind to IREs in target mRNAs to control the expression of key iron-handling proteins [82, 83, 85, 86]. In our models, ferritin levels were particularly dysregulated in G2019S LRRK2 lines, while IRP1/2 activity showed genotype-specific variation. Notably, cytosolic ferritin levels did not correlate strictly with IRP binding activity, implying that lysosomal dysfunction and impaired ferritin turnover may dominate ferritin regulation in the context of mutant LRRK2 signaling. Interestingly, G2019S showed stronger effects on IRP/ferritin regulation compared to R1441C and Y1699C, whereas lysosomal iron was more affected by the non-kinase domain mutants. A recent study Goldman et al. reported impaired regulation of the ferritinophagy adaptor NCOA4 in a homozygous G2019S LRRK2 RAW264.7 macrophage line [121]. Previously, we reported elevated levels of ferritin in the brain of G2019S LRRK2 mice, following proinflammatory stimulation [32]. It is possible that disruption of ferritinophagy in the context of G2019S LRRK2 underlies altered ferritin levels and mobilization, while ROC-COR domain mutations may impact iron homeostasis via partially divergent mechanisms. Also consistent with our findings, Goldman et al. found incomplete rescue of their iron related phenotypes with MLi-2 but improved rescue with a different class of kinase inhibitor [121]. Thus, future work will be required to understand the mechanisms underlying Type I versus Type II LRRK2 kinase inhibitor responses in the context of iron regulation.

Our data reveal that LRRK2 mutations drive lysosomal iron accumulation in both neurons and astrocytes, supporting a model in which iron dysregulation arises through both cell-autonomous and non-cell-autonomous mechanisms. Astrocytes are essential regulators of brain iron homeostasis, not only by sequestering and buffering excess iron, but also by modulating iron availability at synaptic interfaces through secretion of factors that support neuronal iron uptake [41, 42, 45–47, 122]. LRRK2 is expressed in astrocytes [31, 123, 124], and its kinase activity has been reported to modulate astrocytic cytokine release [43, 44, 48]. The increase in lysosomal ferrous iron observed in LRRK2 mutant astrocytes suggests a failure of their iron buffering capacity, that in an in vivo setting would translate into alterations of the synaptic iron microenvironment. In parallel, the accumulation of lysosomal iron in LRRK2 mutant neurons reflects intrinsic defects in iron trafficking and storage, potentially sensitizing neurons to oxidative injury and ferroptosis-related signaling. These findings highlight the potential for both cell-autonomous and non-cell-autonomous disruption of iron handling downstream of pathogenic LRRK2 mutations.

While these findings link pathogenic LRRK2 signaling to iron dyshomeostasis, there are limitations of the present study. The magnitude of the individual phenotypes varied across both mutation and cell type, indicating that the downstream consequences of mutant LRRK2 are not fully uniform. In addition, although our data demonstrate Rab8a as a contributor to iron dysregulation [32], they do not exclude the possibility that other endolysosomal mechanisms are involved. Future studies are needed to define more precisely the trafficking steps linking LRRK2, Rab8a, and iron handling, and determine whether distinct pathogenic LRRK2 mutations act through partially divergent mechanisms. Candidate mechanisms include DMT1 trafficking to lysosomal membranes, turnover of ferritin by ferritinophagy, and lysosomal iron export, each of which could influence intracellular iron availability [23, 125]. Proximity ligation assays or related spatial-proteomic approaches could further test whether pathogenic LRRK2/Rab8a signaling alters the association of iron-handling mediators with lysosomal or recycling compartments. It will also be important to establish whether similar lysosomal iron redistribution occurs in vivo in preclinical models, building on prior studies reporting altered global iron pathways in LRRK2 systems [32, 35, 126]. Additionally, while iron chelation and LRRK2 inhibition both rescued aspects of these phenotypes, a dual therapeutic strategy remains to be tested in preclinical models. Importantly, iron chelation is an effective treatment in Neurodegeneration with Brain Iron Accumulation that often includes parkinsonism symptoms [127–130], but recent clinical trials in PD failed [131] suggesting that targeting precise iron pathways pertaining ferroptosis signaling, in combination with other strategies, in the brain rather than systemic chelation may be considered for future therapeutic efforts for altering disease progression.

## Conclusion

Our findings identify lysosomal iron dysregulation as a key downstream consequence of pathogenic LRRK2 activity in PD. We provide evidence that LRRK2 mutations alter neuronal and glial iron homeostasis, in part through effects on lysosomal iron trafficking, and that disruption of this pathway leads to redox imbalance. Our data further implicate Rab8a as a downstream mediator linking LRRK2 signaling and iron biology, offering insight into how genetic and idiopathic PD may converge on a shared pathogenic pathway.

## Supplementary Information

Below is the link to the electronic supplementary material.

Supplementary Material 1

## Data Availability

Materials and additional details can be made available by the corresponding authors upon request.

## Abbreviations

PD: Parkinson’s disease
iPSC: Induced pluripotent stem cell
iNs: Induced neurons
iAs: Induced astrocytes
DAs: Dopaminergic neurons
LRRK2: Leucine-rich repeat kinase 2
LLOMe: l-leucyl-l-leucine methyl ester
ROS: Reactive oxygen species
FAC: Ferric ammonium citrate
DEF: Deferoxamine
TfR1: Transferrin receptor 1
DMT1: Divalent metal transporter 1
Fth1: Ferritin heavy chain
Ftl: Ferritin light chain
IRP: Iron regulatory protein
IRE: Iron-responsive element
ICP-MS: Inductively coupled plasma mass spectrometry
QSM: Quantitative susceptibility mapping
PPMI: Parkinson’s Progression Markers Initiative
ANOVA: Analysis of variance
WT: Wild-type
TH: Tyrosine Hydroxylase
